# Editorial: Community series in intravital microscopy imaging of leukocytes, volume II

**DOI:** 10.3389/fimmu.2025.1615392

**Published:** 2025-05-09

**Authors:** Connie H. Y. Wong, Craig N. Jenne, Elzbieta Kolaczkowska

**Affiliations:** ^1^ Centre for Inflammatory Diseases, Department of Medicine, School of Clinical Sciences at Monash Health, Monash Medical Centre, Monash University, Clayton, VIC, Australia; ^2^ Department of Microbiology, Immunology and Infectious Diseases, University of Calgary, Calgary, AB, Canada; ^3^ Department of Experimental Hematology, Institute of Zoology and Biomedical Research, Jagiellonian University, Krakow, Poland

**Keywords:** intravital microscopy (IVM), leukocytes, imaging, neutrophils, myloid cells

The field of intravital microscopy (IVM) has come a long way since the method was established as we know it. The first humble attempts from the 19^th^ century, with application of very basic light microscopes, allowed for initial observations of leukocytes in transparent tissues. In fact, these observations resulted in correct and clinically relevant description of a phenomenon known currently as diapedesis: “an emerging of colorless blood corpuscles from the interior of a vein to the outside, completely through the intact wall” (1) albeit the process itself was an enigma at the time. As of late 1970s, the method was revived (2) and gradually improved surgically and microscopically, especially upon the advent of confocal and multi-photon scopes which enabled for detection of fluorescent markers (antibodies, dyes or proteins).

The current volume of the Research Topic was proceed by volume I published in years 2019-2020 (3–13) and is summarized herein (14). The first volume focused on the latest methodological advancements in the field of IVM as well as some novel findings both in terms of unstudied diseases, and leukocyte function and cellular fate. In the current volume the papers continued reporting on the latest developments in the field as well as clinically relevant discoveries accomplished with the past 5 years.

The review paper by Suthya et al. published in our Research Topic, starts by reminding us of the humble beginnings of IVM mentioned above but then updates the reader on the newest hardware, with a particular focus on imaging the brain. The types of microscopes discussed ranged from confocal microscopes through single- and multiphoton devices, leading to photoacoustic imaging (PAI). When it comes to imaging dynamic processes, spinning-disk confocal scopes, or their more contemporary alternates such as resonant-scanning confocal scopes possibly combined with multiphoton imaging, are preferred. The review also discusses the tissue penetration depth that can be achieved with each imaging technology, whilst providing the newest information on labelling tools required for IVM imaging of any organ, and emerging reporter animal models. One of the technical struggles with traditional fluorescent microscopes is the limited number of fluorescent channels, however this has been overcome with spectral scopes (i.e. the above mentioned resonant-scanning platforms) which are capable of detecting any signals in any spectrum, solve the problem. In light of this, there will be an emerging expansion of fluorophores than those existing as technically any wavelength along the spectrum can be detected by these new microscopes. However, they are not accessible to all in the field and Suthya et al. describe how application of some of the newest generations of detectable molecules i.e. quantum dots of nano-crystals/particles, can enable more flexibility even when scope technology is limited. The work-around is further emphasized by Porto-Pedrosa et al., which is introduced in the subsequent paragraphs, underlining the key aspects of new fluorochromes, and in particular demonstrates usefulness of Brilliant Violet and other new generations of polymers.

One of the most extensively investigated leukocytes by IVM are neutrophils. Neutrophils are first responders to the inflammatory insult and can eliminate pathogens by multiple approaches, many of which are linked to their ability to release an array of mediators (some 1300 unique proteins) carried in their numerous granules (15). Pathogen elimination by neutrophils may occur either intracellularly following phagocytosis or extracellularly upon granule release (degranulation) or formation of neutrophil extracellular traps (NETs) (16, 17). Yam et al. focus their review on recent advancements of imaging neutrophils in the context of cancer. When it comes to methodology, the review focuses on appropriate antibodies (e.g. Gr-1 versus Ly6G) and reporter mice that recently revolutionized the IVM field. Also, the review by Suthya et al. underlines the importance of the latter model in brain studies, as staining with antibodies when neutrophils halt blood flow prevents antibody access to these cells thereby limiting labelling, and furthermore, prolonged exposure to anti-Ly6G antibodies can eventually lead to neutrophil depletion. These issues are completely solved when using *Catchup^IVM^* mice, animals expressing genetically-encoded fluorescent reporter molecules within neutrophils. An exciting part of the Yam et al. paper highlights the use of IVM in revealing the contribution of neutrophil in cancer development/metastasis. Here, neutrophils were shown to support transformed cell development, and limit their motility (lower velocity) when inside the tumor microenvironment presumably to interact with cancer cells. Furthermore, NETs can trap circulating tumor cells in hepatic sinusoids, and modulate to the metastatic process. Previously NETs were also show to awaken dormant cancer cells (18).

Intravital imaging of the beating heart is one of the most challenging and recent advances in IVM (19). In this Research Topic, Bumroongthai et al. used this technique in mice to investigate if therapies aimed at preventing damage to coronary microvessels which accompanies opening of occluded coronary arteries; a surgical procedure performed to patients with myocardial infarction. The group reports that they perfected culturing of mesenchymal stromal cells (MSCs) that when introduced into the mouse have the potential to limit this injury. This work involved the 3D culture of cells representing two distinct populations of bone marrow-derived MSCs. One line (CD317neg MSCs-Y201) turned out to be most effective as it limited neutrophil and platelet recruitment, especially when co-applied with heparin. These results carry an important clinical potential.

Another organ that was explored by co-authors of this Research Topic was the spleen. The study by Porto-Pedrosa et al. investigated the dynamics of immune development within this organ across postnatal period. The study reports that although the lymphoid tissue had similar structural arrangements in both newborn mice and adult individuals, they differed in the relative composition (cell number) of some of the myloid cells – macrophages and monocytes, but not neutrophils. The important characteristic of the study was its detailed methodological approach, developing high dimensional intravital microscopy though the use of conventional confocal microscopes working as multi-channel imaging platforms.

The reviews and original papers published in volume II of our Research Topic touch upon historical aspect of IVM studies, refer to the most used techniques and present latest methodological achievements in the field of IVM. The intrinsic technical limitations or unawareness of dye applications, might limit imaging to few channels whereas the newest developments significantly improve this aspect as discussed in the papers. The Research Topic also presents information/work on the heart, spleen and brain imaging, a less frequently studied organs as opposed to well documented liver or cremaster muscle studies, revealing the expansion of potential application of IVM. Importantly, the original research papers reveal results which hold a therapeutic potential. Overall, this Research Topic shows that intravital microscopy stands strong and still carries a potential to bring new discoveries and methodological advancement in the *in vivo* biomedical studies.

## References

[1] Cohnheim J. Ueber entzündung und eiterung. Arch für Pathol Anat und Physiol und für Klin Med. (1867) 40:1–79. doi: 10.1007/BF02968135/METRICS

[2] Granger DN Granger JP Brace RA Parker RE Taylor AE. Analysis of the permeability characteristics of cat intestinal capillaries. Circ Res. (1979) 44:335–44. doi: 10.1161/01.RES.44.3.335 761315

[3] Montague SJ Lim YJ Lee WM Gardiner EE. Imaging platelet processes and function-current and emerging approaches for imaging. Vitro vivo. Front Immunol. (2020) 11:78. doi: 10.3389/fimmu.2020.00078 32082328 PMC7005007

[4] Davis RP Surewaard BGJ Turk M Carestia A Lee WY Petri B. Optimization of *In vivo* Imaging Provides a First Look at Mouse Model of Non-Alcoholic Fatty Liver Disease (NAFLD) Using Intravital Microscopy. Front Immunol. (2019) 10:2988. doi: 10.3389/fimmu.2019.02988 31969883 PMC6960139

[5] Pizzagalli DU Latino I Pulfer A Palomino-Segura M Virgilio T Farsakoglu Y. Characterization of the dynamic behavior of neutrophils following influenza vaccination. Front Immunol. (2019) 10:2621. doi: 10.3389/fimmu.2019.02621 31824481 PMC6881817

[6] Cichon I Ortmann W Bednarz A Lenartowicz M Kolaczkowska E. Reduced neutrophil extracellular trap (NET) formation during systemic inflammation in mice with menkes disease and wilson disease: copper requirement for NET release. Front Immunol. (2020) 10:3021. doi: 10.3389/fimmu.2019.03021 32010131 PMC6974625

[7] Sody S Uddin M Gruneboom A Gorgens A Giebel B Gunzer M. Distinct spatio-temporal dynamics of tumor-associated neutrophils in small tumor lesions. Front Immunol. (2019) 10:1419. doi: 10.3389/fimmu.2019.01419 31293583 PMC6603174

[8] Lopez MJ Seyed-Razavi Y Yamaguchi T Ortiz G Sendra VG Harris DL. Multiphoton intravital microscopy of mandibular draining lymph nodes: A mouse model to study corneal immune responses. Front Immunol. (2020) 11:39. doi: 10.3389/fimmu.2020.00039 32153558 PMC7050419

[9] Jamali A Seyed-Razavi Y Chao C Ortiz G Kenyon B Blanco T. Intravital multiphoton microscopy of the ocular surface: alterations in conventional dendritic cell morphology and kinetics in dry eye disease. Front Immunol. (2020) 11:742. doi: 10.3389/fimmu.2020.00742 32457740 PMC7227427

[10] Ortiz G Chao C Jamali A Seyed-Razavi Y Kenyon B Harris DL. Effect of dry eye disease on the kinetics of lacrimal gland dendritic cells as visualized by intravital multi-photon microscopy. Front Immunol. (2020) 11:1713. doi: 10.3389/fimmu.2020.01713 32903439 PMC7434984

[11] Allan-Rahill NH Lamont MRE Chilian WM Nishimura N Small DM. Intravital microscopy of the beating murine heart to understand cardiac leukocyte dynamics. Front Immunol. (2020) 11:92. doi: 10.3389/fimmu.2020.00092 32117249 PMC7010807

[12] Sedin J Giraud A Steiner SE Ahl D Persson AEG Melican K. High resolution intravital imaging of the renal immune response to injury and infection in mice. Front Immunol. (2019) 10:2744. doi: 10.3389/fimmu.2019.02744 31921099 PMC6916672

[13] Ahl D Eriksson O Sedin J Seignez C Schwan E Kreuger J. Turning up the heat: local temperature control during *in vivo* imaging of immune cells. Front Immunol. (2019) 10:2036. doi: 10.3389/fimmu.2019.02036 31507619 PMC6718468

[14] Wong CHY Jenne CN Kolaczkowska E. Editorial: intravital microscopy imaging of leukocytes. Front Immunol. (2020) 11:2137. doi: 10.3389/fimmu.2020.02137 33013902 PMC7511581

[15] Rørvig S Østergaard O Heegaard NHH Borregaard N. Proteome profiling of human neutrophil granule subsets, secretory vesicles, and cell membrane: correlation with transcriptome profiling of neutrophil precursors. J Leukoc Biol. (2013) 94:711–21. doi: 10.1189/JLB.1212619 23650620

[16] Kolaczkowska E Kubes P. Neutrophil recruitment and function in health and inflammation. Nat Rev Immunol. (2013) 13:159–75. doi: 10.1038/nri3399 23435331

[17] SantockiMSuchADrabDBurczykGKolaczkowskaE. NETs persisting in vasculature undergo self-renewal with consequences for subsequent infection: a mouse model study. Blood. (2025) 145(18):2070–85. doi: 10.1182/BLOOD.2024026643 39841013

[18] Albrengues J Shields MA Ng D Park CG Ambrico A Poindexter ME. Neutrophil extracellular traps produced during inflammation awaken dormant cancer cells in mice. Science. (2018) 361:eaao4227. doi: 10.1126/SCIENCE.AAO4227 30262472 PMC6777850

[19] Li W Nava RG Bribriesco AC Zinselmeyer BH Spahn JH Gelman AE. Intravital 2-photon imaging of leukocyte trafficking in beating heart. J Clin Invest. (2012) 122:2499–508. doi: 10.1172/JCI62970 PMC3386827 22706307

